# Study on the mechanism of SW inhibiting testosterone synthesis in mouse Leydig cells

**DOI:** 10.3389/fvets.2026.1912572

**Published:** 2026-09-01

**Authors:** Zhenxin Chen, Yukai Zhou, Xiaodie He, Jiaming Liu, Yu Zhao, Jiayue Liu, Shenghao Shi, Yucong Gou, Jin Hong, Dongyang Ye, Lei Gao

**Affiliations:** 1 College of Agriculture and Animal Husbandry, Qinghai University, Xining, China; 2 College of Veterinary Medicine and Biosafety, Lanzhou University, Lanzhou, China; 3 College of Veterinary Medicine, Northwest A&F University, Yangling, China

**Keywords:** mice, reproductive toxicity, Swainsonine, testosterone secretion, TM3

## Abstract

**Background:**

Swainsonine (SW), the main toxic component of locoweed, can cause livestock poisoning and reproductive damage in male animals; however, the mechanism by which it affects testosterone secretion remains unclear.

**Methods:**

Ten-week-old male C57BL/6 mice were orally administered SW at doses of 0, 0.05, and 0.25 mg/(kg·d) for 28 days. TM3 mouse Leydig cells were treated with SW at concentrations of 0, 1, and 10 nM for 24 h. The Kyoto Encyclopedia of Genes and Genomes (KEGG) pathway enrichment analysis was performed on RNA-seq data from mouse testicular tissues to identify differentially enriched pathways between the control and SW-treated groups. Testosterone secretion levels were measured using an enzyme-linked immunosorbent assay (ELISA). The mRNA expression levels of steroidogenesis-related genes (*StAR, Cyp11a1, Hsd3b2*, and *Hsd17b3*) were detected by qPCR, while the expression of the steroidogenic acute regulatory (STAR) protein was detected by western blotting. AutoDock Vina molecular docking was used to predict the binding affinity between SW and the STAR protein.

**Results:**

KEGG analysis revealed a significant enrichment of pathways related to steroid synthesis. In both the mouse model and TM3 cells, SW significantly inhibited testosterone secretion, downregulated the mRNA expression of *StAR, Cyp11a1, Hsd3b2*, and *Hsd17b3*, and reduced the protein expression of STAR. Molecular docking analysis revealed multiple potential hydrogen-bond interaction sites between SW and STAR.

**Conclusion:**

SW downregulates the expression of steroidogenesis-related genes and STAR protein, thereby suppressing testosterone secretion in male mice and TM3 cells.

## 1 Introduction

Swainsonine (SW) is a trihydroxy indolizidine alkaloid and the primary toxic component of locoweed. *Oxytropis ochrocephala* (*O. ochrocephala*) Bunge, a common locoweed species distributed in the western grasslands of China, has remarkable adaptability to adverse environmental conditions, including cold temperatures, drought, salinity, alkalinity, and sand storms (1, 2). The common name “locoweed” refers to its toxic effects; ingestion of this plant leads to chronic neurological conditions in animals, which is characterized by a staggering gait and muscular incoordination (3). *O. ochrocephala* has a complex chemical composition that includes flavonoids, saponins, indolizidine alkaloids, quinolizidine alkaloids, and other compounds (2–6). These compounds exhibit diverse biological activities, including insecticidal, cytotoxic and antineoplastic properties (2, 4, 7). Previous studies have confirmed that the ingestion of plants containing SW can cause tissue damage in multiple organs and systems, including the reproductive, nervous, endocrine and immune systems, with the reproductive system being the most severely affected (8–10).

The reproductive performance of livestock has always been a key area of research because it is closely related to population sustainability and economic benefits. Current research indicates that grazing on locoweed leads to substantial early embryonic loss in both cattle and sheep (11). When pregnant ewes forage on locoweed, it causes fetal and placental dysplasia, and it may also trigger abnormal fetal fluid that leads to abortion (12, 13). Studies have demonstrated that rams that consume locoweed experience significant decreases in body weight, thyroxine concentration, testosterone levels, testicular size, and spermatogenesis (14).

Testosterone, secreted by Leydig cells, is a critical hormone in male reproductive physiology. It plays an essential role in the development and maintenance of secondary sexual characteristics and fertility. It is synthesized from cholesterol, which is transported into mitochondria by steroidogenic acute regulatory (STAR) protein, via a series of sequential enzymatic reactions catalyzed by cytochrome P450 family 11 subfamily A member 1 (CYP11A1), hydroxy-delta-5-steroid dehydrogenase, 3-beta delta-isomerase 2 (HSD3B2), and hydroxysteroid 17-beta dehydrogenase 3 (HSD17B3) (15). Research has shown that decreased testosterone levels can lead to a variety of adverse health outcomes, including sexual dysfunction, reduced energy levels, decreased muscle mass, mood disorders, altered bone mineral density, cardiovascular complications, depression, diminished sexual desire, erectile dysfunction, reduced ejaculatory volume, and loss of body and facial hair (16, 17).

Testosterone is essential for male physiological functions, and it is well documented that the consumption of locoweed suppresses testosterone secretion. However, the specific causative agent responsible for this effect has not yet been confirmed. Accordingly, this study was designed to assess the effect of SW, the primary toxic component of locoweed, on testosterone production in mouse testes and TM3 Leydig cells, with the aim of exploring the molecular basis underlying the observed reduction in testosterone secretion.

## 2 Materials and methods

### 2.1 Animals

All animal-handling procedures were approved by the Ethics Committee of Qinghai University, Qinghai, China (Approval No. SL-2022002). A total of 18 pubertal male C57BL/6J mice (6 weeks old) were purchased from Beijing Vital River Laboratory Animal Technology Co., Ltd. (Beijing, China). After arrival and before the experiment, all mice were acclimated to the animal facility for 1 month to eliminate the effects of transportation and were housed under controlled temperature (22 °C−23 °C) and a standard 12-h light/12-h dark cycle. According to previous reports (8), mice were randomly divided into three groups with six mice in each group, and they received SW at doses of 0, 0.05, and 0.25 mg/(kg·d) by daily oral gavage. After 28 days, blood and testicular tissue samples were collected from the anesthetized mice. The testes were immediately snap-frozen in liquid nitrogen and then transferred to an ultra-low temperature freezer at −80 °C for storage until further analysis.

### 2.2 Cell cultures

TM3 cell lines (Mouse Leydig cells, ATCC CRL-1714, Washington, USA) were used in this experiment. The cells were cultured in DMEM-F/12 (DF12, Gibco, Carlsbad, CA, USA) supplemented with 10% fetal bovine serum (FBS, Gibco) and 1% antibiotic-antimycotic (AA, Invitrogen, Carlsbad, CA, USA), and maintained in a 37 °C humidified atmosphere containing 5% CO_2_.

### 2.3 Measurement of cell proliferation and viability

TM3 cell viability was measured using a Cell Counting Kit-8 assay (CCK-8, Beyotime, Shanghai, China). Briefly, TM3 cells were seeded at a density of 2 × 10^3^ cells per well in 96-well plates. The cells were treated with 100 μL of SW at gradient concentrations (0, 0.1, 1, 10, 100, 1,000 nM) for 24 h. After treatment, 10 μL of CCK-8 solution was added to each well, and the plates were incubated for an additional 2 h at 37 °C in a humidified 5% CO_2_ atmosphere. We compared the relative optical density of each treatment group to that of the untreated control group. Based on the assay results, 1 nM and 10 nM SW were selected for subsequent cell-based experiments, aligning with the treatment concentrations used in previous studies.

### 2.4 RNA-sequencing

Testicular tissues were collected from the NC group and the daily 0.25 mg/(kg·d) SW-treated group, with six biological replicates per group. Transcriptome sequencing was conducted by Beijing CapitalBio Technology with the Illumina NovaSeq 6,000 platform. After differential gene screening, Kyoto Encyclopedia of Genes and Genomes (KEGG) enrichment analysis and intergroup difference analysis were performed.

### 2.5 Testosterone assay

Blood was collected from the mice, and after 2 h of rest, serum was separated by centrifugation at 2,000 r/min for 10 min. Culture supernatants were collected at 24 h after SW treatment. Next, testosterone concentration was measured using commercial enzyme-linked immunosorbent assay (ELISA) kits (ABclonal, China) following the manufacturer's instructions. Fluorescence intensity was measured using a microplate reader at a wavelength of 450 nm. The minimum detectable concentration of testosterone was 0.02 ng/mL. The intra-assay and inter-assay coefficients of variation were less than 10% and 15%, respectively, and three biological replicates were performed for each treatment in each assay.

### 2.6 RNA extraction and real-time quantitative PCR (qPCR)

Testis tissue and TM3 cell samples from different treatment groups were collected, and total RNA was extracted using the TRIzol method (TaKaRa, Japan). The extracted RNA samples were diluted with RNase-free water (TianGen, China), and OD values and total RNA concentrations were measured using a biospectrophotometer (Thermo Fisher, USA). RNA integrity was detected via 2% agarose gel electrophoresis using a gel imaging system (Thermo Fisher, USA). A total of 1 μg of each qualified RNA sample was taken for reverse transcription into cDNA using the TaKaRa kit (TaKaRa, Japan). Appropriate qPCR primers were designed according to the mRNA sequences of steroidogenesis-related genes, and the primer sequences are shown in Table 1. The 20 μL reaction system used a SYBR (Synergetic binding reagent) Green Mix kit (TaKaRa, Japan), and samples mixed on ice were transferred away from light to the QuantStudio 6 Flex system (Thermo Fisher, USA); all reactions were performed in accordance with the operating instructions. Melting curve analysis was conducted after the reaction to verify the specificity of amplified products. Based on the Ct values of samples, relative quantification was performed using the 2^−ΔΔ*Ct*^ method, with *36b4* as the reference gene. The effects of SW on the transcription levels of steroidogenesis-related genes were analyzed and compared.

**Table 1:** Primer sequences.

Names	GenBank accession no.	Primer sequences (5^′^-3^′^)	Product size (bp)
*StAR*	NM_011485.5	F:TAAACTCACTTGGCTGCTCAGTATTG	101
		R:GGTGGTTGGCGAACTCTATCTG	
*Hsd3b2*	NM_001359741.1	F:CCAGGGCATCTCTGTTGTCAT	105
		R:GGTTCTGGGTACCTTTCAGATTGA	
*Cyp11a1*	NM_019779.4	F:AGGTCCTTCAATGAGATCCCTT	137
		R:TCCCTGTAAATGGGGCCATAC	
*Hsd17b3*	NM_008291.3	F:ATGAGCCCGTTTGCCTCTG	244
		R:CCACAGGTAACAAGTCTTGGTC	
*36b4*	NM_007475.5	F:CTCACTGAGATTCGGGATAAG	223
		R:CTCCCACCTTGTCTCCAGTC	

### 2.7 Protein extraction and western blotting (WB)

Total protein was extracted from testicular tissue and TM3 cells with RIPA (Radio immunoprecipitation assay) lysis buffer (Thermo Fisher, USA). Equal volumes of total cellular proteins were resolved using 10% sodium dodecyl sulfate-polyacrylamide gel electrophoresis after determining the protein concentration using the BCA (Bicinchoninic acid) method and diluting to the corresponding concentration, and electrophoretically transferred onto PVDF (Polyvinylidene difluoride) membranes (Thermo Fisher, USA). After blocking with 10% skim milk (Solarbio, China) dissolved in Tris-buffered saline containing 0.1% Tween 20 (TBST) at 25 °C for 2 h, the membranes were incubated with primary antibodies diluted in TBST overnight at 4 °C, followed by incubation with horseradish peroxidase (HRP)-conjugated secondary antibodies diluted in TBST at 4 °C for 2 h. Peroxidase activity was detected with an ECL hypersensitive solution kit (Thermo Fisher, USA). Immunoreactive bands were imaged with a Gel Image system (Bio-Rad Laboratories, USA) and digitized with Quantity One software (Bio-Rad Laboratories, USA). The primary antibodies used were rabbit anti-STAR (A22166, ABclonal, China; 1:1,000) and mouse anti-β-actin (TA-09, Zhongshan Jinqiao, China; 1:2,000). The secondary antibodies used were HRP-conjugated goat anti-rabbit (Zhongshan Jinqiao, China; 1:2,000) and HRP-conjugated goat anti-mouse (Zhongshan Jinqiao, China; 1:2,000).

### 2.8 Molecular docking

The crystal structures of the STAR candidate protein targets (PDB ID: 1JSS) were retrieved from the RCSB Protein Data Bank (https://www.rcsb.org/) and subsequently modified using Autodock Tools 1.5.6 software: Scripps Research Institute, La Jolla, CA, USA, which involved removing ligands and water molecules, adding hydrogen atoms, and assigning Gasteiger partial charges. The 3D structural files of the SW (PubChem CID: 51683) were obtained from the PubChem database (https://pubchem.ncbi.nlm.nih.gov/). The structural files were imported into ChemBio3D software and subjected to energy minimization using the MMFF94 force field to relieve spatial strain. All files were saved in the “PDBQT” format and subsequently imported into AutoDock Vina 1.2.0 for global blind docking, with the docking box defined by the following parameters: center_x = 41.699, center_y = 9.705, center_z = 26.956; dimensions: size_x = 47.25, size_y = 47.25, size_z = 46.5; and the grid spacing is 0.375 Å. The exhaustiveness parameter was set to 128 to improve the completeness of conformational sampling. After docking is complete, the software automatically outputs binding poses ranked by binding free energy from lowest to highest. Generally, a binding energy below −5 kcal/mol is accepted as the criterion for a potentially stable interaction. The five conformations with the lowest binding energies were selected as candidate binding poses, and for each conformation, the number of hydrogen bonds, bond lengths (in Å), and the involved amino acid residues were recorded. Finally, the molecular docking results were visualized using PyMOL software (18).

### 2.9 Data and statistical analyses

All data are expressed as the Mean ± SD. Statistical analyses were performed using one-way analysis of variance (ANOVA) with GraphPad Prism 7.0: GraphPad Software San Diego, CA, USA. Transcriptome sequencing data were analyzed using R 4.2.2: R Foundation for Statistical Computing, Vienna, Austria. (R Foundation for Statistical Computing). Differences were considered significant at a *P*-value of < 0.05. Statistical power analysis was conducted to calculate the number of animals required to detect significant differences.

## 3 Results

### 3.1 Transcriptome sequencing revealed that SW impairs steroid hormone biosynthesis in male mice

**Figure 1:**
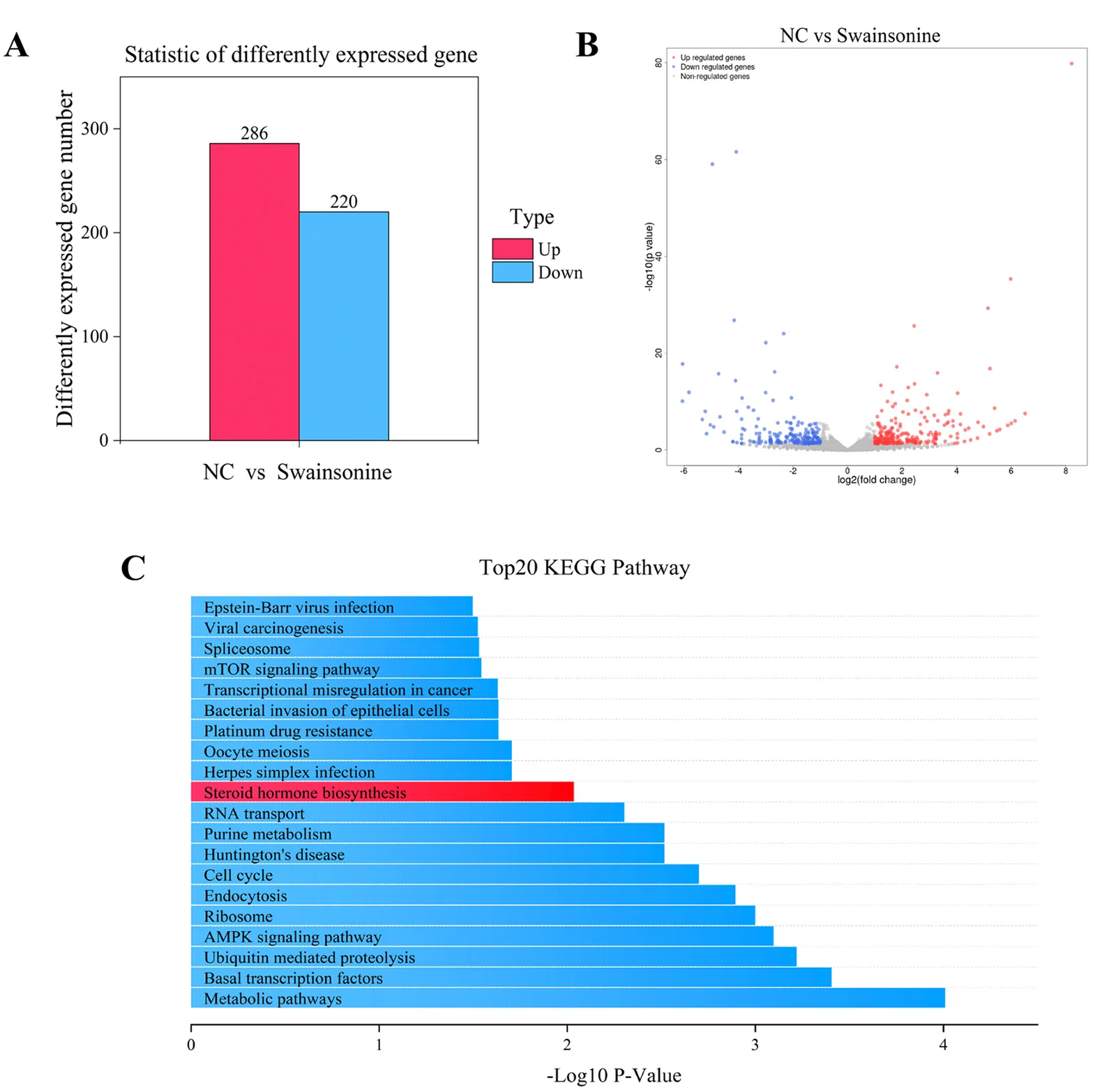
Differential gene expression in the testicles of mice. Testicular tissues were obtained from the NC group and mice fed with SW at a dose of 0.25 mg/(kg·d). **(A)** Numbers and **(B)** volcano plots of differential genes in the testis. **(C)** Top 20 KEGG pathways enriched in differential genes in the testis (*n* = 6).

To elucidate the impact of SW on testicular function in male mice, transcriptomic profiling was performed on testicular tissues obtained from both the control and SW-treated groups. Differentially expressed genes (DEGs) in mouse testes were identified based on fold change (log2FC > 1.0) and significance (*P* < 0.05). As shown in the statistical bar chart (Figure 1A), a total of 506 DEGs were identified: after SW treatment, 286 genes were significantly upregulated, and 220 genes were downregulated. The volcano plot (Figure 1B) further illustrates the expression variation and statistical significance of all detected genes: red dots represent significantly upregulated DEGs, blue dots indicate significantly downregulated DEGs, and gray dots denote genes with no significant difference in expression. We then performed KEGG enrichment analysis on these DEGs, and the top 20 most significantly enriched pathways are shown in Figure 1C, covering key research areas including energy metabolism, autophagy, the cell cycle, and the mTOR and AMPK signaling pathways. Steroid hormones secreted by the testes are critical reproductive hormones in male animals, playing pivotal roles in population reproduction and economic productivity. Accordingly, we focused our analysis on the steroid hormone biosynthesis pathway. KEGG enrichment analysis showed that the DEGs in testes were mainly enriched in the steroid hormone biosynthesis pathway (Figure 1C). Among these DEGs, the upregulated genes included *Cyp1b1, Cyp2c29, Cyp2e1*, and others, while the downregulated genes included *H2-Ke6, Cyp21a1, Hsd3b6*, and others.

### 3.2 SW inhibited testosterone levels and the expression of steroidogenic genes and protein in male mice

**Figure 2:**
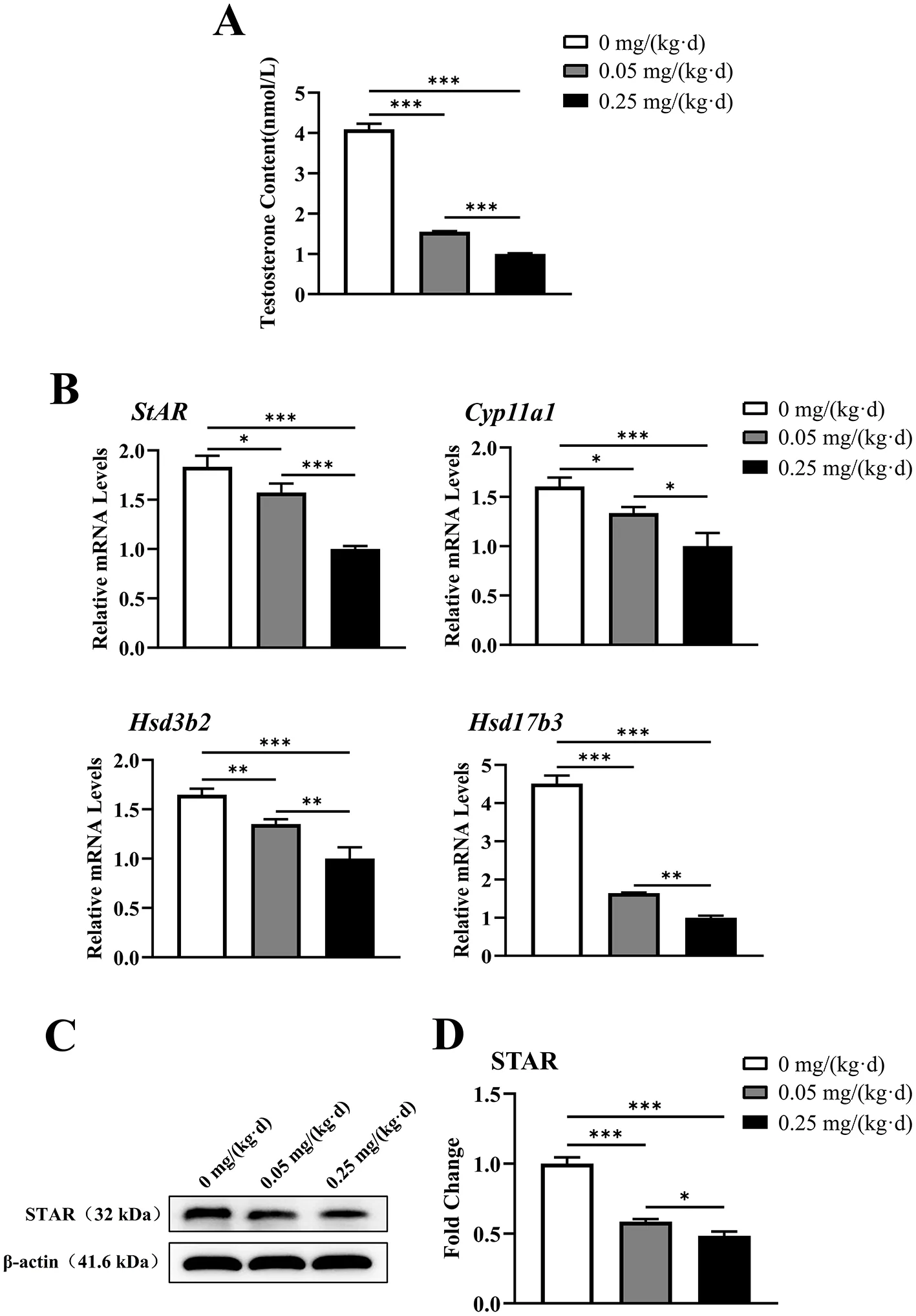
Effect of Swainsonine on testosterone synthesis in mice. Testes and serum samples were collected from mice in the control group, low-dose group, and high-dose group that were fed SW (0, 0.05, and 0.25 mg/(kg·d), respectively) after the completion of the modeling period. **(A)** Serum testosterone levels were measured using an ELISA kit. **(B)** Total RNA was extracted from testis tissues, and mRNA levels of steroidogenesis-related genes were quantified by qPCR. **(C)** STAR levels were examined through western blotting. **(D)** The ratio of the levels of STAR and β-actin was determined from the densities of the immunoreactive bands. Data shoulder mark * indicates significant difference (*P* < 0.05), ** indicates significant difference (*P* < 0.01), *** indicates significant difference (*P* < 0.001), and no asterisk indicates no significant difference (*P* > 0.05); The data are displayed as mean ± standard deviation (*n* = 3).

To confirm the findings from transcriptome sequencing, serum testosterone levels were quantified by ELISA in the NC group and in groups treated with SW at doses of 0.05 mg/(kg·d) and 0.25 mg/(kg·d). As shown in Figure 2A, compared with the NC group, serum testosterone levels in the 0.05 mg/(kg·d) and 0.25 mg/(kg·d) groups were significantly decreased (*P* < 0.001). Additionally, serum testosterone in the 0.25 mg/(kg·d) group was significantly lower than that in the 0.05 mg/(kg·d) group (*P* < 0.001). Testosterone is synthesized from steroid precursors through a cascade of reactions mediated by steroidogenic genes including *StAR, Cyp11a1, Hsd3b2*, and *Hsd17b3*. In the 0.05 mg/(kg·d) SW-treated group, the expression levels of *StAR* (*P* < 0.05), *Cyp11a1* (*P* < 0.05), *Hsd3b2* (*P* < 0.01), and *Hsd17b3* (*P* < 0.001) were significantly lower than those in the NC group. Notably, compared with the NC group, 0.25 mg/(kg·d) SW significantly suppressed the relative expression of all four genes, with all differences reaching statistical significance (*P* < 0.001). Furthermore, compared with the 0.05 mg/(kg·d) group, the 0.25 mg/(kg·d) group showed significant decreases in the expression of *StAR* (*P* < 0.001), *Cyp11a1* (*P* < 0.05), *Hsd3b2*, and *Hsd17b3* (*P* < 0.01), as presented in Figure 2B. WB analysis further corroborated this trend, demonstrating a significant reduction in STAR protein levels in the testes of mice treated with 0.05 mg/(kg·d) SW (*P* < 0.001) and 0.25 mg/(kg·d) SW (*P* < 0.001) compared with the NC group. The STAR protein level in the 0.25 mg/(kg·d) group was also significantly lower than that in the 0.05 mg/(kg·d) group (*P* < 0.05), as shown in Figures 2C, D. We therefore conclude that SW reduces testosterone secretion in mice by downregulating steroidogenesis-related genes and proteins.

### 3.3 SW inhibited testosterone levels and the expression of steroidogenic genes and proteins in TM3 cells

**Figure 3:**
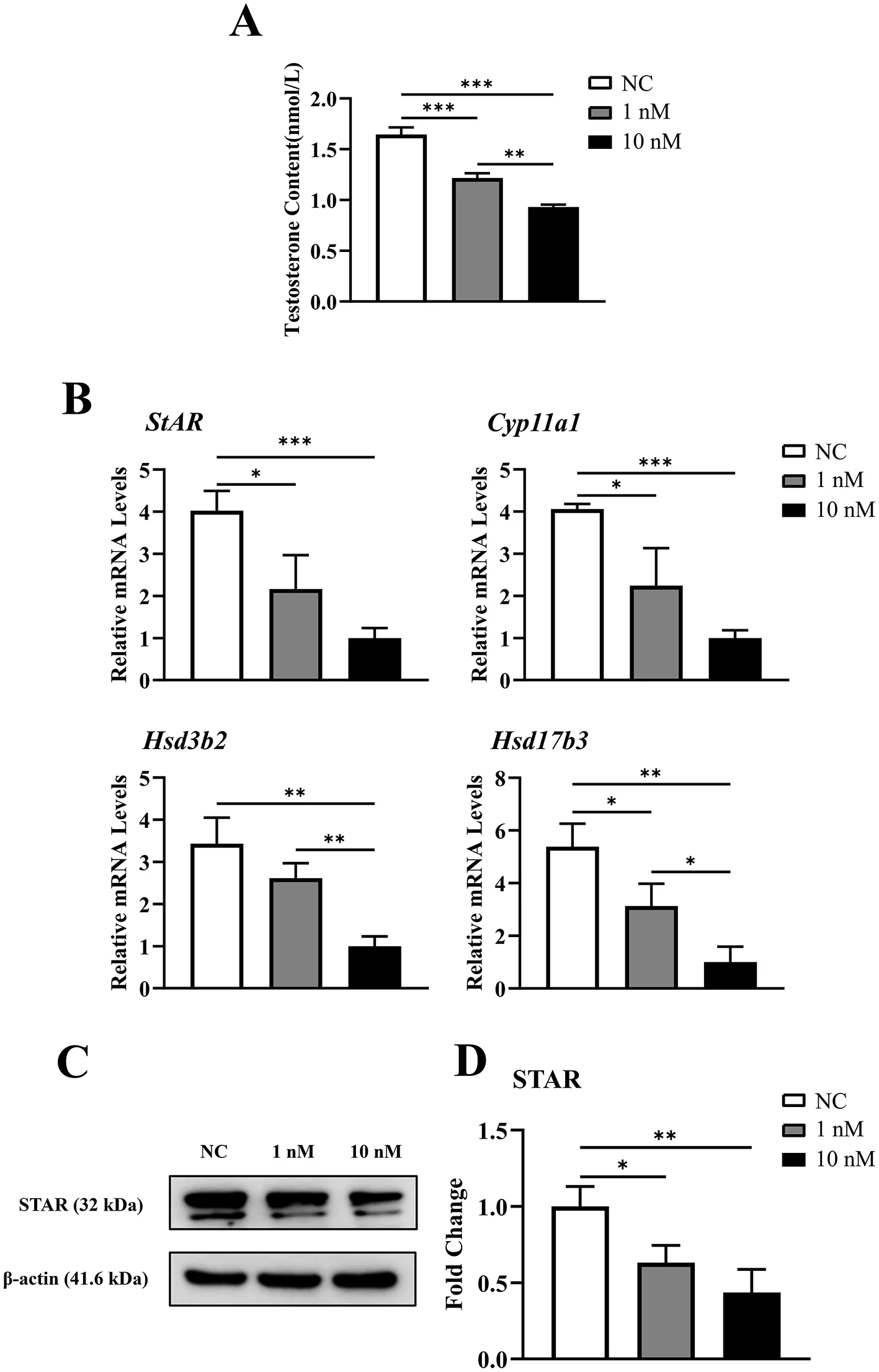
Effect of Swainsonine on testosterone synthesis in TM3. **(A)** The supernatant testosterone levels were measured using an ELISA kit. **(B)** Total RNA was extracted from TM3 cells, and the mRNA levels of steroidogenesis-related genes were quantified by qPCR. **(C)** Representative images of STAR protein levels detected by western blotting. **(D)** The ratio of the levels of STAR and β-actin was determined from the densities of the immunoreactive bands. Data marked with * indicate significant difference (*P* < 0.05), ** indicates significant difference (*P* < 0.01), *** indicates significant difference (*P* < 0.001), and no asterisk indicates no significant difference (*P* > 0.05). The data are displayed as mean ± standard deviation (*n* = 3).

TM3 cells, derived from mouse Leydig cells, stably express genes involved in steroid synthesis and secrete testosterone, making them a valuable tool for investigating testosterone secretion. Based on cell viability results (Supplementary Figure S1), we selected 1 and 10 nM as the optimal concentrations for treating TM3 cells with SW to conduct subsequent experiments. Supernatant and TM3 cells were collected after 24 h of treatment with SW at 0 nM (NC), 1 nM, or 10 nM. Compared to the NC group, testosterone levels in the supernatant were significantly reduced in the 1 nM (*P* < 0.01) and 10 nM (*P* < 0.001) groups, with the 10 nM group showing a statistically greater reduction in testosterone than the 1 nM group (*P* < 0.001) (Figure 3A). The expression of genes related to steroidogenesis was detected. The results showed that compared to the NC group, significant reductions in *StAR, Cyp11a1*, and *Hsd17b3* were observed in the 1 nM group (*P*<*0.05*), while highly significant reductions in *StAR* (*P* < 0.001), *Cyp11a1* (*P* < 0.001), *Hsd3b2* (*P* < 0.01), and *Hsd17b3* (*P* < 0.01) were found in the 10 nM group. Additionally, compared with the 1 nM group, significant decreases in *Hsd3b2* (*P* < 0.01) and *Hsd17b3* (*P* < 0.05) were observed in the 10 nM group (Figure 3B). The STAR protein levels showed a similar trend. Compared with the NC group, STAR protein levels were significantly decreased in the 1 nM group (*P* < 0.05) and the 10 nM group (*P* < 0.01). Furthermore, STAR protein levels were lower in the 10 nM group than in the 1 nM group, although this difference did not reach statistical significance (Figures 3C, D). Collectively, these findings demonstrate that SW exposure modulates STAR protein expression levels in TM3 cells in a concentration-dependent manner. These data suggest that SW reduces testosterone secretion in TM3 cells by downregulating steroidogenesis-related genes and the STAR protein.

### 3.4 Molecular docking predicted a potential interaction between SW and the STAR protein

**Figure 4:**
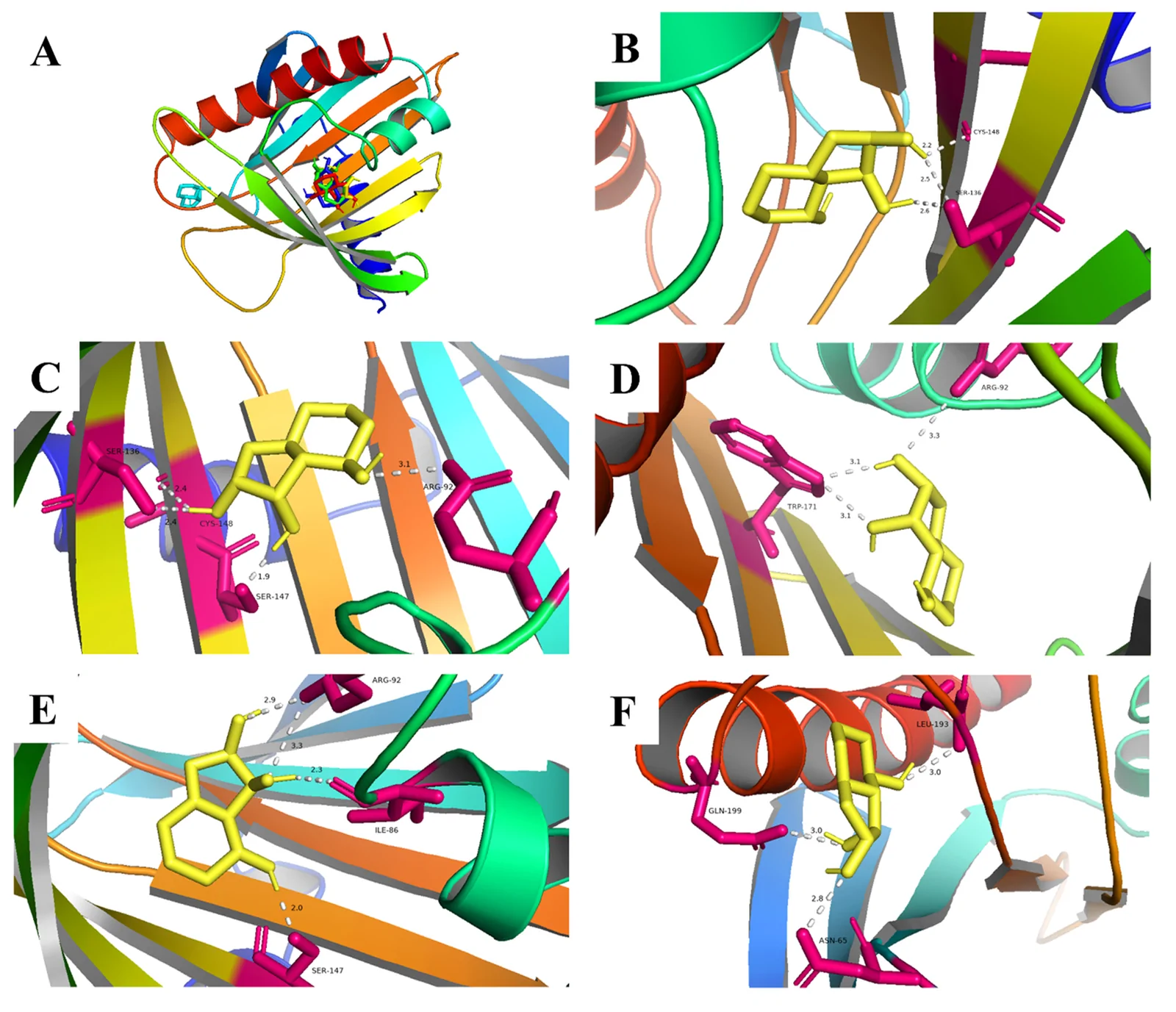
Computational simulation of the binding modes between Swainsonine and the STAR protein was visualized. **(A)** The general locations of the top five binding sites with the highest binding energies are displayed as follows: Top1 in red, Top2 in yellow, Top3 in blue, Top4 in green, and Top5 in cyan. **(B–F)** The schematic illustrates Top1–Top5, where yellow represents Swainsonine, pink denotes the residues interacting with Swainsonine, and their names are labeled adjacent to them. The white connecting lines between pink and yellow indicate hydrogen bonds, with the numerical values above the lines representing the bond lengths (Unit: Ångströms, Å).

In this study, molecular docking was performed between SW and STAR to investigate the mechanism by which SW affects STAR. The top five binding sites with the highest affinity are presented, where lower binding energy indicates stronger binding capacity. The predicted binding energies of SW and STAR were all below −5 kcal/mol (Table 2), suggesting a strong binding potential. SW was found to interact with STAR through multiple hydrogen bonds, with key binding residues including Ser^136^, Ser^147^, Arg^92^, and Cys^148^ (Table 2 and Figure 4). Therefore, SW is might to interact with the STAR protein through this potential binding mode.

**Table 2:** Molecular docking results of Swainsonine with the STAR protein were obtained.

Binding site	Connected residues	Hydrogen bond number	Docking scores (kcal/Mol)
1	SER^136^, CYS^148^	3	−6.2
2	ARG^92^, SER^136^, SER^147^, CYS^148^	4	−6.1
3	ARG^92^, TRP^171^	3	−5.8
4	ILE^86^, ARG^92^, SER^147^	4	−5.6
5	ASN^65^, LEU^193^, GLN^199^	3	−5.6

## 4 Discussion

SW is a characteristic toxic alkaloid produced by locoweeds, including plant species in the genus *Oxytropis*. When livestock ingest locoweed over a long period, it causes multi-system damage to the nervous, endocrine, and reproductive systems. A typical symptom of intoxication in male animals is reduced testosterone levels and impaired reproductive performance. Previous studies have only observed decreased serum testosterone in rams fed roughage containing locoweed, but could not distinguish the effects of individual plant components (13). To date, the molecular mechanism by which the SW monomer regulates testosterone synthesis in male animals remains unclear. In this study, we established both a subchronic *in vivo* exposure model using adult male mice and an *in vitro* model using TM3 mouse Leydig cells. By integrating testicular transcriptome sequencing, quantification of key steroidogenic molecules, and AutoDock Vina molecular docking, we systematically elucidated the complete molecular pathway through which SW inhibits testosterone secretion.

After 28 days of feeding, we collected testes from mice in the NC group and the 0.25 mg/(kg d) SW-treated group and performed RNA transcriptome sequencing. The results showed that a large number of differentially expressed genes were identified in mouse testes between the control and SW-treated groups. KEGG enrichment analysis revealed significant differences in the steroid biosynthesis pathway, mTOR signaling pathway, and AMPK signaling pathway, all of which play critical roles in male reproduction. Among the enriched genes, *Hsd3b6*, a member of the 3β-hydroxysteroid dehydrogenase family that catalyzes a key step in steroid synthesis (19), was significantly downregulated. This indicates that *Hsd3b6* is the primary target of the steroidogenic process in SW-induced testicular injury.

Testosterone, the primary steroid secreted by the testes, is critical to male reproductive health and serves as a core indicator of male reproductive potential. It plays an essential role in driving male genital development and facilitating (20), stimulates the development of secondary sexual characteristics that help males compete for mates and attract (21–23) partners, and supports mating-related behaviors by boosting libido and aggression in reproductive contexts (24, 25). Previous studies have found that testosterone levels decrease in rams after ingestion of locoweed (14). Our study further demonstrated that SW reduces testosterone levels in both mice and TM3 Leydig cells in a dose-dependent manner, confirming that SW acts directly on Leydig cells rather than indirectly inhibiting testosterone secretion through the hypothalamic-pituitary-testicular axis (26). Gonadotropin-releasing hormone (GnRH), secreted by the hypothalamus, stimulates the pituitary gland to synthesize and release luteinizing hormone (LH) (20, 27). LH binds to the luteinizing hormone receptor on Leydig cells and promotes testosterone biosynthesis by enabling STAR to transport cholesterol into mitochondria. Cholesterol is rapidly converted to pregnenolone by the CYP11A1 enzyme located on the inner mitochondrial membrane. Pregnenolone then enters the endoplasmic reticulum, where a series of enzymatic reactions catalyzed by HSD3B2 and HSD17B3 ultimately convert it into testosterone (15).

Previous studies have reported similar regulatory patterns. Fluoride simultaneously downregulates *StAR, Cyp11a1, Hsd3b*2, *and Hsd17b3*, which is consistent with the toxicity pattern of SW (27, 28). Arsenic epigenetically inhibits *StAR* through histone acetylation (29). Protective trace elements (selenium and silicon), upregulate the complete set of steroidogenic genes and completely antagonize the inhibitory effects of SW (30, 31). Trace mineral elements and plant-derived alkaloid toxins exert bidirectional, opposing regulatory effects on Leydig cell function (32). Locoweed fed to cows temporarily alters ovarian function, increases estrous cycle length, alters reproductive behavior, reduces conception rates, and leads to birth defects in offspring (33). SW can induce apoptosis in goat luteal cells and trophoblast cells, interfere with progesterone and estradiol secretion, and cause estrus disorders, embryo implantation failure, and abortion in female livestock (9, 34, 35). Consistent with previous studies, our study found that SW reduces testosterone secretion and downregulates the expression levels of *StAR, Cyp11a1, Hsd3b2*, and *Hsd17b3* mRNA, as well as STAR protein in mice and mouse Leydig cells. These findings demonstrate that SW inhibits testosterone secretion by suppressing the expression of steroidogenesis-related genes and proteins in mouse testes.

To further explore the underlying molecular mechanism, we conducted molecular docking simulations. The results showed that SW can form hydrogen bonds with residues such as Ser^136^, Arg^92^, and Cys^148^ of the STAR protein. The binding energies of the top five binding conformations were all less than −5 kcal/mol, indicating a stable binding potential. STAR is a nuclear-encoded, mitochondria-targeted protein. Following its phosphorylation and activation in the cytoplasm at human Ser^195^ or murine Ser^194^ and Ser^232^, STAR translocates into mitochondria (36–38). Within mitochondria, STAR facilitates cholesterol transport to the inner mitochondrial membrane, where it is converted to pregnenolone, the precursor for all steroid hormones. However, unphosphorylated STAR in the cytoplasm is rapidly degraded (39). Based on these findings and our sequencing results, we hypothesize that SW binds to STAR and inhibits its phosphorylation-dependent activation, which reduces the efficiency of cholesterol transport. Additionally, SW may also suppress *StAR* gene transcription by disrupting testicular transcriptome signaling (through the mTOR/AMPK pathway), ultimately leading to decreased mRNA synthesis (40). However, molecular docking is only a static computational simulation and cannot replicate the dynamic physiological conditions inside cells, such as intracellular pH, accessory proteins, and the lipid microenvironment. At present, this conclusion remains only a theoretical inference and requires direct biochemical verification through assays such as SPR and cellular STAR overexpression rescue experiments.

This study is the first to demonstrate that the SW is the core toxic substance responsible for locoweed-induced testosterone reduction and reproductive decline in males. This finding clarifies the difference between the combined toxicity of multiple locoweed components and the independent reproductive damage caused by SW. From a grazing management perspective, breeding males, including rams and bulls, must be strictly managed to prevent them from ingesting locoweed. Even long-term exposure to low doses of SW can persistently suppress testosterone secretion, impair mating ability, and ultimately reduce the reproductive efficiency of an entire herd. This study also defines clear targets for developing detoxification agents for locoweed poisoning and protective additives for male reproduction. The reproductive toxicity data obtained from multiple dose gradients in this study can provide toxicological support for hazard classification of locoweed in western grasslands and the formulation of regional grazing management standards, which in turn will help reduce economic losses in pastoral livestock production.

**Figure 5:**
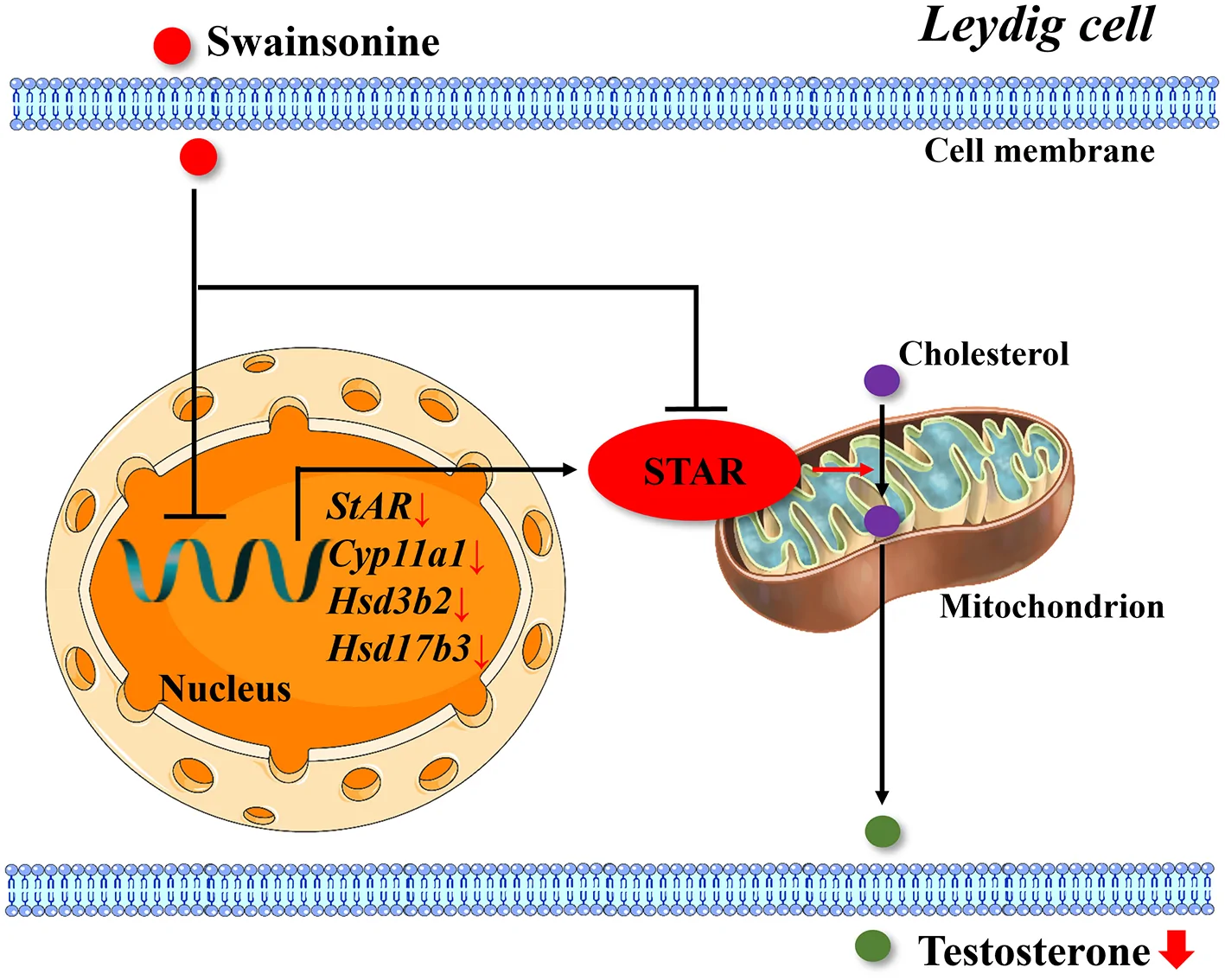
Diagram of the pathway by which Swainsonine regulates testosterone secretion in male mice.

We identified key differentially expressed genes in the testes of SW-exposed mice, analyzed their enriched pathways, and paid special attention to pathways related to steroid synthesis. Our experimental results confirmed that SW suppresses testosterone secretion in both mice and TM3 cells. Furthermore, SW may suppress the phosphorylation-dependent activation of STAR, consequently blocking cholesterol translocation into mitochondria. In conclusion, this study demonstrates that SW inhibits testosterone secretion by downregulating the expression of steroidogenesis-related genes and the STAR protein in mouse Leydig cells (Figure 5).

## Data Availability

The data presented in the study are deposited in the RCSB Protein Data Bank (https://www.rcsb.org/)(accession number 1JSS) and the PubChem database (https://pubchem.ncbi.nlm.nih.gov/) (accession number 51683).
